# Harnessing the tissue and plasma lncRNA-peptidome to discover peptide-based cancer biomarkers

**DOI:** 10.1038/s41598-019-48774-1

**Published:** 2019-08-23

**Authors:** Sajib Chakraborty, Geoffroy Andrieux, A. M. Mahmudul Hasan, Musaddeque Ahmed, Md. Ismail Hosen, Tania Rahman, M. Anwar Hossain, Melanie Boerries

**Affiliations:** 1 Molecular systems biology laboratory, Department of Biochemistry and Molecular Biology, University of Dhaka, Dhaka, Bangladesh; 2 Institute of Medical Bioinformatics and Systems Medicine, Medical Center - University of Freiburg, Faculty of Medicine, University of Freiburg, Freiburg, Germany; 3 German Cancer Consortium (DKTK) and German Cancer Research Center (DKFZ), Partner Site Freiburg, Freiburg, Germany; 4 Princess Margaret Cancer Centre/University Health Network, Toronto, Ontario Canada

**Keywords:** Cancer prevention, Data processing, Proteome informatics

## Abstract

Proteome-centric studies, although have identified numerous lncRNA-encoded polypeptides, lack differential expression analysis of lncRNA-peptidome across primary tissues, cell lines and cancer states. We established a computational-proteogenomic workflow involving re-processing of publicly available LC-MS/MS data, which facilitated the identification of tissue-specific and universally expressed (UExp) lncRNA-polypeptides across 14 primary human tissues and 11 cell lines. The utility of lncRNA-peptidome as cancer-biomarkers was investigated by re-processing LC-MS/MS data from 92 colon-adenocarcinoma (COAD) and 30 normal colon-epithelium tissues. Intriguingly, a significant upregulation of five lncRNA UExp-polypeptides in COAD tissues was observed. Furthermore, clustering of the UExp-polypeptides led to the classification of COAD patients that coincided with the clinical stratification, underlining the prognostic potential of the UExp-polypeptides. Lastly, we identified differential abundance of the UExp-polypeptides in the plasma of prostate-cancer patients highlighting their potential as plasma-biomarker. The analysis of lncRNA-peptidome may pave the way to identify effective tissue/plasma biomarkers for different cancer types.

## Introduction

Deep sequencing of human transcriptome has identified numerous long transcribed RNA molecules lacking conserved open reading frames (ORFs)^1^. The term long non-coding RNAs (lncRNAs) was coined to specify these long RNA molecules of over 200 nucleotides since these RNA molecules are thought not to encode polypeptides or proteins^2^. The number of lncRNA transcripts in human is almost three times larger than the number of protein coding mRNAs^3^. Although the protein coding genes are well characterized, detailed annotations, categorization and tissue-specific expression profiles of lncRNAs are still emerging. GENCODE consortium^4^ adopted a strategy by combining manual annotation and EnSEMBL based annotation^5^ to categorize lncRNAs. According to GENCODE categorization, lncRNAs can be sub-grouped depending on their genomic-locations with respect to protein-coding genes into different biotypes: Antisense RNA, Long intergenic noncoding RNA (LincRNA), Sense-overlapping, Sense-intronic and Processed transcript^4^. Recently, attempts were undertaken to determine the tissue-specificity of lncRNA transcripts. For instance Jiang *et al*. identified 1,184 ubiquitously expressed and 2,583 tissue-specific lncRNAs by analyzing sixteen RNA-seq datasets across human tissues from various independent studies^6^. Although the study lacked the analysis of cell lines and disease states such as cancer, nevertheless highlighted many important features of these ubiquitously expressed lncRNAs including conserved exons and/or promoter sequences^6^.

LncRNAs are involved in diverse biological processes ranging from genomic imprinting and organization of chromosome structure^7^ to regulating enzymatic activity^8^. Moreover differential expression of lncRNAs has been associated with cell-fate programming/reprogramming^9^ and diseases^10^. The capacity of lncRNA transcripts to alter or modify cellular machinery boosted the hypothesis that lncRNAs may also be linked with human diseases. For instance It was reported that aberrant regulation of lncRNA transcripts is associated with the progression of different cancers^11^. The lncRNA - Hox transcript antisense intergenic RNA (HOTAIR) exhibited elevated expression levels in primary and metastatic breast tumors and showed the promise to be a predictor for breast cancer metastasis and mortality^12^. Later the higher HOTAIR expression level in different cancer tissues including colorectal^13^, cervical^14^, lung^15^ and pancreatic^16^ cancers compared to normal counterpart strengthened the notion that this can be a universal biomarker for cancer diagnosis and prognosis^17^.

With ever increasing aging population, cancer incidence and mortality rates are increasing at an alarming rate^18^. In this desperate situation, identification of novel cancer-biomarkers facilitating better patient stratification, early diagnosis and prognosis are of utmost importance. The utility of lncRNA transcripts as biomarkers seems to be challenging because of their variable stability^19^. Unstable nature of lncRNAs may hinder the possibility of their usage as biomarkers. In a genome-wide study involving the mouse Neuro-2a cell line, Clark *et al*. compared the half-lives of 12,000 cellular mRNAs with wide range of lncRNAs and showed that lncRNAs are less stable than mRNAs on an average^19^. The utility of lncRNAs as biomarkers has recently drawn much attention because of the identification of extracellular circulating lncRNAs for heart failure^20^, kidney^21^ and coronary artery diseases^22^. In a recent study, Schlosser *et al*. attempted to investigate 84 highly abundant lncRNAs via RT-qPCR in plasma samples from pulmonary arterial hypertension (PAH) patients^23^. Surprisingly the majority of lncRNA molecules including previously reported circulating lncRNA biomarker candidates were undetectable in plasma whereas most of these lncRNAs were readily detectable in pulmonary tissue^23^.

Recent studies have shown that lncRNA transcripts physically interact with ribosomes in human cell line^24^, mouse embryonic stem cell^25^, zebrafish^26^ and arabidopsis^27^. These findings have not only revealed the evolutionary conserved nature of ribosome binding property of the lncRNAs but also challenged the previous notion that lncRNAs lack coding potential. In a proteome-centric study conducted by Wilhelm *et al*. provided evidence for 430 small polypeptides encoded by lncRNAs by analyzing mass-spectrometry data from human tissues^28^. Corroborating these findings Verheggen *et al*. found mass-spectrometric evidence for 1% of lncRNA transcripts in the database LNCipedia (https://lncipedia.org/)^29^. Few studies have already proved the benefit of re-processing the Mass-spectrometric (LC-MS/MS) raw data to search for hidden lncRNA encoded polypeptides^29,30^. However, whether the abundances of lncRNA polypeptides akin to their corresponding RNA transcripts are tissue specific or ubiquitously expressed remains unresolved. Moreover differential regulations of lncRNA encoded polypeptides in primary tissues, cell lines and cancer state are unknown. In this manuscript, we sought to investigate the lncRNA encoded polypeptides in primary human tissues, cell lines, cancer tissues and plasma samples. We first established a computational proteogenomic workflow that was used to re-process the mass-spectrometric (LC-MS/MS) raw files for the identification and quantification of the lncRNA polypeptides in human tissues and cell lines. The primary objective was to quantify the abundance lncRNA polypeptides across different human tissues and cell lines to determine whether they are tissue specific or universally expressed. Furthermore we extended the study to investigate the differential abundance of lncRNA polypeptides between cancer tissue/plasma samples and their normal counterparts. To this end, large number of publicly available LC-MS/MS raw files comprising tissue/plasma samples from colon adenocarcinoma (COAD) and prostate cancer patients along with their normal counterparts were retrieved and reprocessed by the custom-built computational workflow. In summary, we aimed to unravel the cancer-driven differential abundance of lncRNA encoded peptides in normal and cancer tissues. The outcome of the study can have far reaching effect on our understanding of lncRNA biology in carcinogenesis and more importantly it may pave a unique path to identify lncRNA derived peptide-based biomarkers for cancer diagnosis and prognosis.

## Results

### Establishment of an integrated proteogenomic workflow for the identification and quantification of lncRNA encoded polypeptides

**Figure 1:**
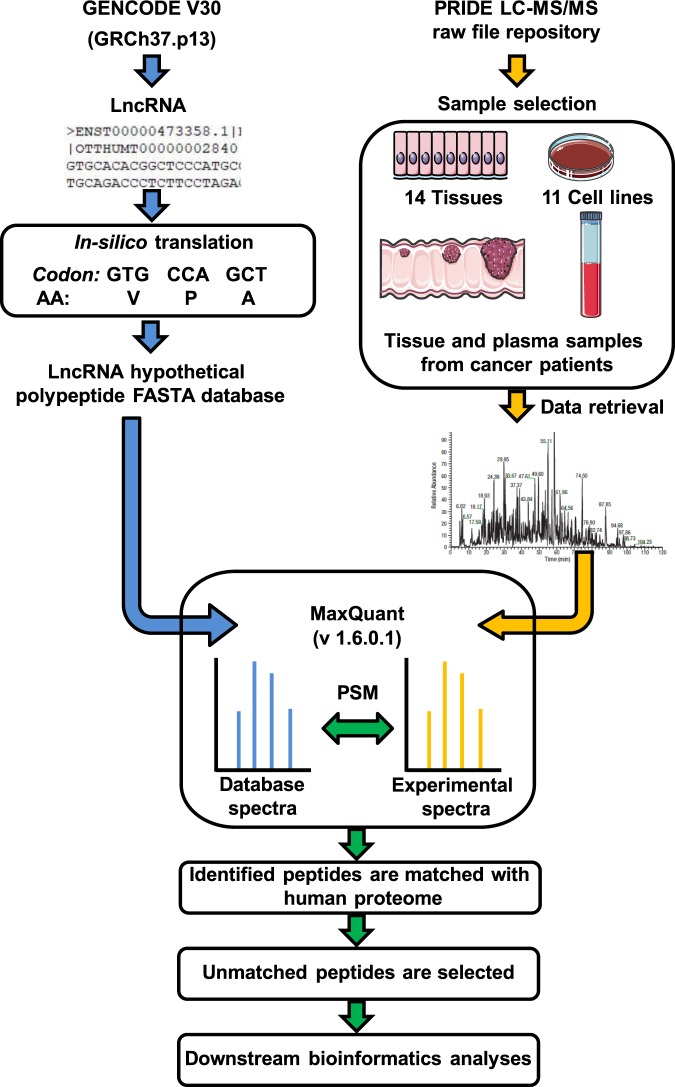
Integrated computational workflow. Integrated computational workflow was established which is divided into multiple segments. First step was to retrieve the nucleotide sequences of 23,898 long non-coding RNA (lncRNA) transcripts from GENCODE V30 (GRCh37.p13) followed by the in-silico three-frame translation to obtain the hypothetical polypeptide sequences encoded by lncRNA transcripts to assemble a FASTA database encompassing the hypothetical polypeptide sequences from lncRNA transcripts. Additionally canonical human reference proteome sequences were also integrated into this custom-built FASTA database. Secondly, LC-MS/MS raw files originating from 14 human tissues, 11 cell lines, 92 Colon cancer (COAD) samples, 30 normal colon samples, prostate cancer tissues and adjacent tumor-free tissues from three patients and plasma samples from prostate cancer patients and healthy individuals were retrieved from PRIDE database. The LC-MS/MS raw files were subjected to MaxQuant (Version 1.6.0.1) for re-processing. Custom built FASTA database was used for peptide spectrum match (PSM) in Andromeda searching. The Identified lncRNA peptides were matched to human proteome and only the unmatched fractions of the peptides were retained. Finally these peptides/polypeptides were used for downstream bioinformatics analysis.

The preliminary goal of this study was to investigate the coding potential of lncRNAs by quantifying the abundance of the lncRNA encoded polypeptides (or lncRNA-polypeptides in short). To achieve that, we developed an integrated proteogenomic pipeline to identify and quantify the polypeptides as the translated products of the lncRNA transcripts in the human genome (Fig. 1). In short, we first predicted the hypothetical polypeptide sequences from each of the lncRNA transcripts annotated in the human genome. We then exhaustively searched for these hypothetical polypeptides in the LC-MS/MS spectra to identify polypeptides that are actually translated in human primary tissues and cell lines. To avoid any remote chance of miss-annotation we removed any peptides that matched with human proteome and the remaining unmatched peptides were selected for further analyses.

PRoteomics IDEntifications (PRIDE) database (https://www.ebi.ac.uk/pride/archive/) was mined systematically to extract the mass-spectrometry (LC-MS/MS) raw files representing 14 different human tissues, 11 cell lines, 92 colon adenocarcinoma (COAD), 30 normal colon epithelium, three prostate cancer tissues along with their adjacent tumor-free histologically normal tissues, plasma samples from two prostate cancer patients and two healthy subjects (Supplementary Table S1). The extracted LC-MS/MS raw files were then subjected to MaxQuant (Version: 1.6.0.1) re-processing using an in house custom-built FASTA database representing human proteome in addition to lncRNA-peptidome as background database for peptide spectrum match (PSM). The criteria for minimum number of peptides required to report an lncRNA hit was set as 2 implying that at least two non-overlapping unique peptides were required per lncRNA-polypeptide to be quantified. Analysis of the lncRNA-peptides revealed that the relative abundances of the different peptides encoded by a given lncRNA transcript in a particular tissue follow a similar abundance pattern. To demonstrate this, abundance profiles peptides encoded by two exemplary lncRNAs - LINC00969 and RP11-203J24.9T, are shown in Fig. S1A,B. Peptides encoded by either of these lncRNAs displayed a similar abundance pattern with low coefficient of variation (%CV) in a particular tissue. On the contrary, same peptide belonging to a particular lncRNA transcript exhibited variable abundance across the tissues. The variability of lncRNA-peptide abundance was significant across tissues as revealed by the ANOVA test (p = 0.001) (Fig. S1A,B). These findings suggested that the factor influencing the variability of lncRNA peptides abundance-profiles was the tissue of origin rather than the sequence and length of the peptides. Overall these results showed the robustness of the lncRNA peptide quantification. The label free quantification (LFQ) intensity representing the abundance of lncRNA polypeptides were calculated as the median of all peptide intensities corresponding to a given lncRNA. Since at least two non-overlapping unique peptides were identified for each lncRNA transcript, the translational products of the lncRNAs were considered as polypeptides rather than peptides.

### Identification of tissue-specific and ubiquitously expressed lncRNA polypeptides in primary human tissues

**Figure 2:**
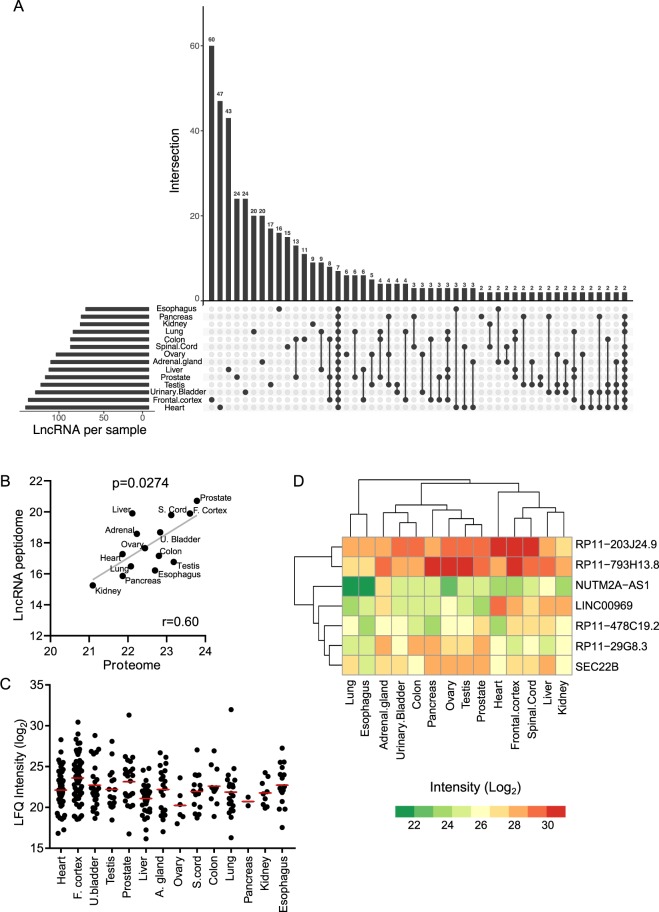
Characterization of lncRNA-peptidome in primary human tissues. (**A**) Identification of the tissue-specific and ubiquitously expressed lncRNA polypeptides. The horizontal bars represent the total number of lncRNA polypeptides identified in each human tissue samples. The vertical bars represent the number of polypeptides identified in a single (tissue-specific) or multiple tissues. The dots indicate the tissues for which the polypeptides were identified. (**B**) Correlation plot of average intensity (Log_2_) of all identified proteins (proteome) and lncRNA polypeptides (lncRNA-peptidome) across 14 tissues. X- and Y- axis represent the global intensities of proteome and lncRNA-peptidome respectively. Spearman correlation coefficient was calculated and indicated by r. The diagonal line was fitted with the linear regression. P-value represents the statistical significance as calculated by t-test. (**C**) Label free intensities (LFQs) of tissue-specific lncRNA polypeptides across 14 tissues are shown in Log_2_ scale. Each dot represents the LFQ of a particular tissue-specific lncRNA polypeptide. The average LFQ value for each tissue is indicated by red bar. (**D**) Heatmap representing the abundance profile of 7 ubiquitously expressed lncRNA polypeptides across 14 human tissues. The columns (tissues) and rows (LncRNA polypeptides) were clustered by using hierarchical clustering algorithm. The color code represents the intensities of the polypeptides where red and green means high and low intensities, respectively.

In order to investigate abundance profiles of the lncRNA polypeptides, the intensities of the lncRNA polypeptides across 14 tissues were taken into account. A total of 2606 peptides constituting 665 polypeptides encoded by human lncRNA transcripts were identified across 14 different human tissues. This number indicated that almost 3% of the total lncRNA transcriptome is engaged in the translational process to synthesize polypeptides in contrary to the previous report where it was assumed that only 1% of the lncRNA transcripts encode polypeptides^29^. The annotations of the identified lncRNA transcripts with coding potential in the tissues are given in Supplementary Table S2. The categorization of lncRNA-polypeptides based on the genomic location of the corresponding lncRNA transcripts as described recently by Gagliardi *et al*.^31^ revealed that the majority of polypeptides (49%) were encoded by lincRNAs followed by anti-sense lncRNAs (37%), processed transcripts (9%), sense-intronic (3%) and sense-overlapping (2%). The total number of lncRNA encoded polypeptides was highest in heart tissue (n = 137) followed by frontal cortex (n = 134) while the lowest number was for esophagus (n = 70) (Fig. 2A). In order to gain insight about the tissue specificity, we identified the unique polypeptides (designated as tissue-specific) that are solely specific to one particular tissue. For instance, frontal cortex, heart, and liver tissues were found to harbor relatively higher number of tissue-specific lncRNA polypeptides (60, 47 and 43, respectively) that were exclusively expressed in these tissues. For prostate, urinary bladder, lung, adrenal gland and testis tissues, a moderate number of exclusive polypeptides (24, 24, 20, 20 and 17, respectively) were identified. The rest of the tissues (esophagus, kidney and pancreas) expressed relatively lower number of exclusive lncRNA encoded polypeptides (Fig. 2A). On the contrary, seven lncRNA polypeptides (LINC00969, NUTM2A-AS1, RP11-203J24.9, RP11-29G8.3, RP11-478C19.2, RP11-793H13.8, SEC. 22B) showed ubiquitous expression throughout all 14 human tissues, hence considered as ubiquitously expressed lncRNA polypeptides (Fig. 2A).

Next we wanted to investigate whether the abundance profile of lncRNA-peptidome are comparable with that of human proteome across the tissues. The computational workflow established in the current study enabled the simultaneous comparison of human proteome with lncRNA-peptidome by utilizing of a custom built FASTA database in which hypothetical lncRNA-peptidome is merged with canonical human proteome (Fig. 1). This database was particularly useful to quantify the lncRNA polypeptides as well as proteins by re-processing the same set of LC-MS/MS raw files in a single MaxQuant run. The comparison of the global abundance profiles of human proteome and lncRNA-peptidome revealed that, although comparable, the average intensities of tissue-proteomes were significantly higher than that of lncRNA-peptidomes in all the analyzed tissues (Fig. S1C). The highest abundance of proteome and lncRNA-peptidome was observed for prostate followed by liver, frontal cortex and spinal cord (Fig. S1C). On the contrary kidney exhibited the lowest proteome and lncRNA-peptidome abundance (Fig. S1C). Interestingly the global abundance profiles (average intensities) of proteomes and lncRNA-peptidomes across 14 tissues were positively correlated with a relatively higher correlation coefficient value (r = 0.60, p-value = 0.0274) (Fig. 2B). The correlation plot showed a higher global abundance of lncRNA-peptidome and proteome in prostate, frontal cortex and spinal cord. In contrast a lower global abundance of proteome and lncRNA-peptidome was observed in kidney. The concordant global intensities of human proteome and lncRNA-peptidome suggested that there may be a pervasive factor such as translational rate that may contribute to the correlated proteome and lncRNA-peptidome abundance profiles. Previously it was proposed that the translational rate is the dominant factor that may determine the cellular abundance of proteins^32^.

Having established the abundance profiles of lncRNA-peptidome across tissues, we wanted to investigate the abundance profiles of the lncRNA encoded tissue-specific polypeptides across tissues. The tissue-wise comparison revealed a differential expression of the tissue-specific lncRNA polypeptides rather than a uniform abundance profile across 14 tissues (Fig. 2C). The highest mean abundance of lncRNA polypeptides was found in frontal cortex followed by prostate. The lowest mean abundance was observed in ovary (Fig. 2C). Next we sought to investigate the abundance profile of the seven polypeptides (LINC00969, NUTM2A-AS1, RP11-203J24.9, RP11-29G8.3, RP11-478C19.2, RP11-793H13.8, SEC. 22B) that were expressed throughout all 14 human tissues (Fig. 2A,D). Abundance profiling revealed that these seven ubiquitously expressed lncRNA polypeptides are differentially expressed across the tissues. For instance, RP11-203J24.9 and RP11-793H13.8 polypeptides were highly expressed in almost all the tissues, while the abundance level of NUTM2A-AS1 polypeptide was relatively lower in all the tissues (Fig. 2D). The remaining LINC00969, RP11-29G8.3, SEC. 22B and RP11-478C19.2 polypeptides showed a mixed pattern implying a high to moderate abundance in some while lower abundances in the rest of the tissues (Fig. 2D).

### Determination of cell-line specific and ubiquitously expressed lncRNA polypeptides in 11 human cell lines

**Figure 3:**
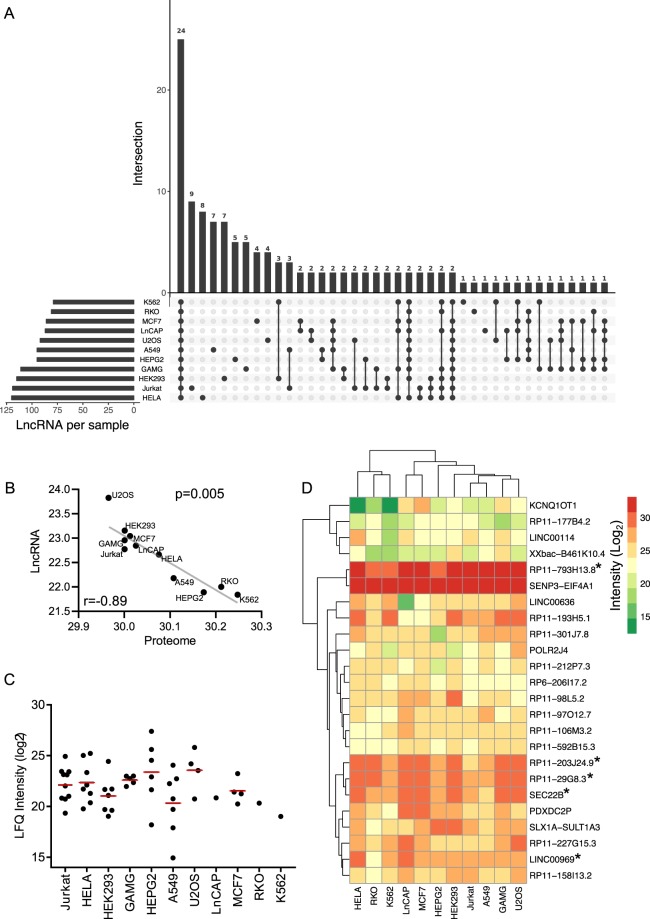
Characterization of lncRNA-peptidome in 11 human cell lines. (**A**) Identification of the cell-line specific and ubiquitously expressed lncRNA polypeptides across 11 human cell lines. The horizontal bars represent the total number of lncRNA polypeptides identified in each cell line whereas the vertical bars represent the number of polypeptides identified in a single (cell line-specific) or multiple cell lines. The dots indicate the cell lines for which the polypeptides were identified as indicated by a bar. (**B**) Correlation plot of average intensity (Log_2_) of all identified proteins (proteome) and lncRNA polypeptides (lncRNA-peptidome) across 11 cell lines. X- and Y- axis represent the global intensities of proteome and lncRNA-peptidome respectively. Spearman correlation coefficient was calculated and indicated by r. The diagonal line was fitted with the linear regression. P-value shows the statistical significance as calculated by t-test. (**C**) Label free intensities (LFQs) of cell line-specific lncRNA polypeptides across 11 tissues are shown in Log_2_ scale. Each dot represents the LFQ of a particular cell line-specific lncRNA polypeptide. The average LFQ value for each tissue is indicated by red bar. (**D**) Heatmap representing the abundance profile of 24 ubiquitously expressed lncRNA polypeptides across 11 cell lines. The columns (cell lines) and rows (LncRNA polypeptides) were clustered by using hierarchical clustering algorithm. The color code represents the intensities of the polypeptides where red and green means high and low intensities, respectively. UExp polypeptides were indicate by star (*).

The presence of lncRNA-peptidome in human tissues encouraged us to investigate the lncRNA-peptidome in human cell lines. To achieve this, LC-MS/MS data from 11 different human cell lines were analyzed for the identification of lncRNA polypeptides. The description of these 11 cell lines including tissue of origin is given in Table 1. A total 256 polypeptides were detected as the translational product of lncRNA transcripts. The annotations of the identified lncRNA transcripts with coding potential in the cell lines are given in Supplementary Table S3. Similar to the tissues, the categorization of cell-line lncRNA polypeptides showed that the majority of polypeptides (47%) belong to lincRNAs followed by anti-sense lncRNAs (31%), processed transcripts (19%), sense-intronic (1.6%) and sense-overlapping (1.6%). The most striking difference of cell line derived lncRNA-peptidome in comparison to that of primary tissues is the opposing number of unique (tissue/cell-line specific) and ubiquitously expressed polypeptides (Fig. 3A). In contrast to tissues, low number of unique (cell-line specific) polypeptides was identified in the cell lines. For example only five cell-line specific polypeptides were identified in GAMG (representing brain tissue) compared to 61 unique lncRNA polypeptides identified in frontal cortex. The highest number of unique polypeptides (n = 9) was identified in Jurkat (Acute T-Cell Leukemia) followed by in HELA (Cervical carcinoma) (n = 8). The most interesting aspect is the higher number (n = 24) of ubiquitously expressed lncRNA polypeptides in the cell lines compared to the tissues (n = 7). Altogether, higher number of ubiquitously expressed and lower number of unique polypeptides in the cell lines, hint toward a more uniform nature of the cell lines in terms of lncRNA-peptidome. Previously it was shown that the cell lines, despite of their distinctive tissue origins are unexpectedly similar with respect to their proteome abundances^33^. To broaden this hypothesis we compared the abundance profiles of proteome and lncRNA-peptidome across 11 cell lines. Intriguingly, akin to the proteomes, lncRNA-peptidome of the 11 cell lines exhibited a striking uniformity, which was absent in case of primary tissues (Fig. S2A). We extended the hypothesis of global uniformity of the cell lines by corroborating that the similarity of cell lines can also be reflected in their respective lncRNA-peptidomes.

**Table 1:** Description of 11 cell lines.

Cell line	Origin
A549	Lung carcinoma
GAMG	Glioblastoma
HEK293	Embryonic kidney cells
Hela	Cervical carcinoma
HepG2	Hepatoma
Jurkat	Acute T-Cell Leukemia
K562	Chronic Myeloid Leukemia
LnCap	Prostate carcinoma
MCF7	Mammary carcinoma
RKO	Colon carcinoma
U2OS	Osteosarcoma

Investigation of the abundance correlation between proteome and lncRNA-peptidome across cell lines surprisingly revealed a strong negative correlation (Fig. 3B). For instance highest lncRNA-peptidome and lowest proteome abundance profiles were observed for U2OS (Osteosarcoma) cell line. Whereas K562 (Chronic Myeloid Leukemia) cell line showed an opposite trend demonstrating lowest lncRNA-peptidome and highest proteome abundance profiles. Since only small numbers of cell-line specific polypeptides were identified, we were not able to draw any conclusion from their abundance profiles although the dynamic-ranges appeared to be similar to the one observed in tissues (Fig. 3C). The abundance comparison of the 24 ubiquitously expressed lncRNA polypeptides showed differential abundance pattern across the cell lines (Fig. 3D). Some of the polypeptides such as RP11-793H13.8 were highly expressed across all cell lines. Clustering analysis of the 24 ubiquitous lncRNA polypeptides yielded two distinct clusters based on their expression. By comparing the overlapping ubiquitously expressed lncRNA polypeptides in tissues (n = 7) and cell lines (n = 24), five lncRNA polypeptides (RP11-793H13.8, RP11-203J24.9, SEC. 22B, LINC00969, RP11-29G8.3) were determined as universally expressed (UExp) (indicated by star in Fig. 3D) in all 14 tissues and 11 cell lines. Four lncRNA polypeptides (KCNQ1OT1, RP11-177B4.2, LINC00114 and XXbac-B461K10.4) showed lower abundances across all the cell lines whereas rest of the lncRNA polypeptides showed moderate to high abundance. Especially two lncRNAs polypeptides including one UExp RP11-793H13.8 and one non-UExp SENP3-EIF4A1 exhibited the highest abundance followed by three UExp polypeptides - RP11-203J24.9, RP11-29G8.3 and SEC. 22B in all cell lines.

### Tissue-matched analysis of lncRNA-peptidome in tissues and cell lines

In order to gain deeper insight into the concordance and discordance between human tissues and cell lines with respect to lncRNA-peptidome, we matched tissues to the corresponding cell lines according to the tissue-origin of the cell lines. In total the five tissue-cell pairs were identified: Lung-A549, Colon-RKO, Liver-HEPG2, Prostate-LnCAP, Frontal cortex-GAMG. The highest percentage of lncRNA-peptidome overlap between a particular tissue-cell line pair was observed for colon-RKO (14.3%) (Fig. S2B) followed by prostate-LnCAP (13.4%) (Fig. S2C), Frontal cortex-GAMG (11.9%) (Fig. S2D) and Liver-HepG2 (10.8%) (Fig. S2E). The lowest overlap was identified for Lung-A549 pair (9%) (Fig. S2F). Total number, as well as number of tissue-specific lncRNA polypeptides was observed to be higher in tissues in comparison with their cell line counterparts (Fig. S2B–F). To compare the lncRNA-peptidome as a fraction of the proteome between matched tissue-cell line pairs, the number of lncRNA polypeptides was divided by the total number of proteins in each of the matched tissue and cell lines and expressed as a fraction of total proteome. The correlation analysis revealed that fraction of proteome representing the lncRNA-peptidome is highly correlated (R = 0.831 with p value of 0.02) between tissue and cell-lines pairs (Fig. S2G). The lowest fraction was observed for colon tissue-RKO pair (0.0113 for colon tissue and 0.0152 for RKO), whereas the highest fraction was monitored for frontal-cortex-GAMG pair (0.015 for frontal cortex and 0.019 for GAMG) followed by Liver-HepG2 pair (0.014 for liver tissue and 0.018 for HePG2) (Fig. S2G).

**Figure 4:**
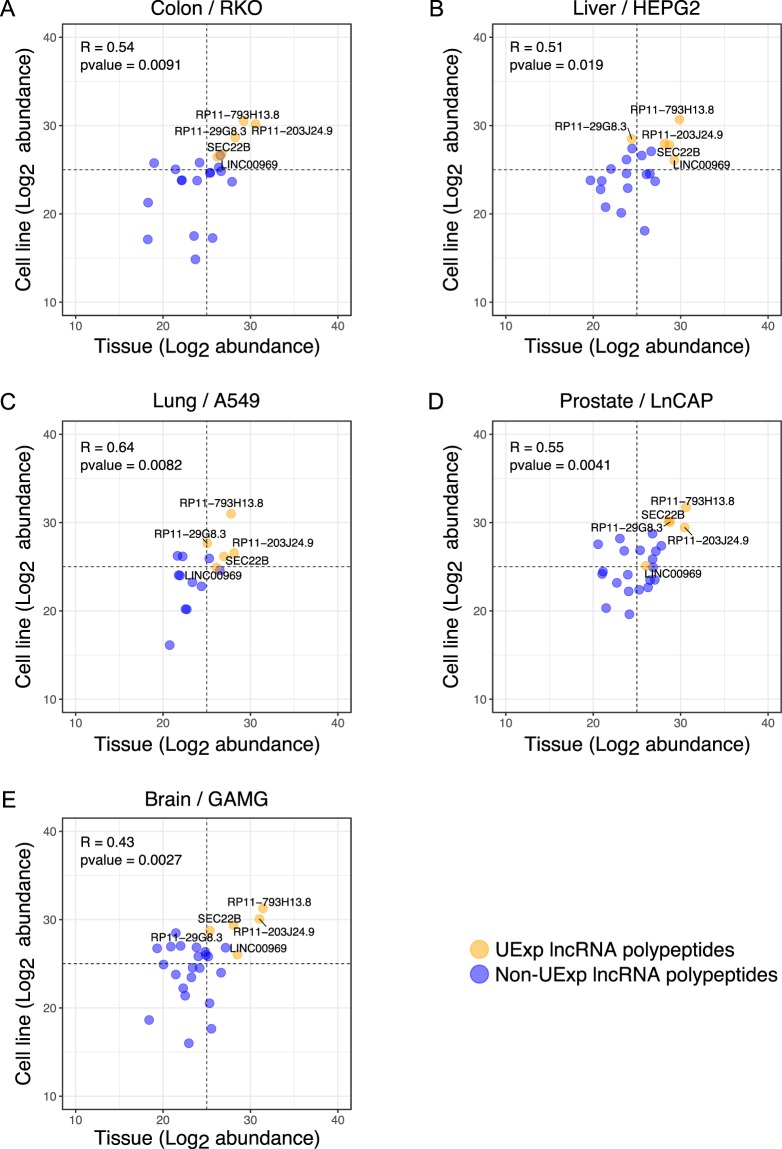
Correlation of common lncRNA polypeptide abundance in tissue-cell line pairs. Tissues and cell lines are matched according to their tissue origin in five pairs: Colon-RKO (**A**), Liver-HepG2 (**B**), Lung-A549 (**C**), Prostate-LnCAP (**D**), and Brain-GAMG (**E**). The scatter plot represents the abundance of overlapping lncRNA polypeptides in a particular tissue-cell line pair. The Pearson correlation coefficient (R) is shown on top left corner for each plot. X-axis and Y-axis represent the abundance (Log_2_) lncRNA polypeptides in the tissue and cell line, respectively. The five ubiquitously expressed lncRNA polypeptides (expressed in all tissues and cell lines) are indicated by orange color whereas the non-ubiquitous polypeptides are marked by blue color. Horizontal and vertical dashed lines divided the plot area into four quadrants where the top right and bottom left quadrants represent higher and lower abundance of lncRNA polypeptides in the corresponding tissue and cell line, respectively. P-value represents the statistical significance of each correlation plot as calculated by t-test.

Next we analyzed the abundance profile of the five UExp lncRNA polypeptides (RP11-793H13.8, RP11-203J24.9, SEC. 22B, LINC00969, RP11-29G8.3). Although these polypeptides were found in all the analyzed tissues and cell lines, whether their abundance profiles are similar between a tissue and its cell line counterpart was investigated next. To this end the abundances of common lncRNA polypeptides including the five UExp-polypeptides in a tissue-cell line pair were plotted (Fig. 4). The correlation plot showed that indeed the abundances of the common lncRNA polypeptides in a tissue-cell line pair were high to moderately correlated (Fig. 4). The highest correlation (person correlation) coefficient was observed for Lung-A549 (0.64) followed by prostate-LnCAP (0.55), colon-RKO (0.54) and liver-HepG2 (0.50), whereas the brain-GAMG pair showed the lowest correlation (0.42) (Fig. 4). All the correlations were found to be statistically significant as revealed by t-test (Fig. 4). The UExp-polypeptides (orange circles) tend to cluster in the top right quadrant indicating their relatively high abundances in tissues and cell lines alike (Fig. 4). MS/MS spectra representing the Individual peptides detected as part of UExp-polypeptides were further analyzed to confirm their validity as true peptides (Fig. S3). The sequences of the peptides identified for each of the five UExp-polypeptides are listed in Supplementary Table S4.

### Determination of prognostic value of lncRNA polypeptides in colon cancer

To determine applicability of lncRNA polypeptides as potential biomarkers in cancer context, the differential abundance of the lncRNA polypeptides in cancer and normal tissues has to be investigated. To achieve this, we retrieved LC-MS/MS raw files from clinically annotated colorectal adenocarcinoma (COAD) and normal colon epithelium tissues as deposited in Clinical Proteomic Tumor Analysis Consortium (CPTAC) (https://proteomics.cancer.gov/programs/cptac). Although apart from COAD, proteogenomic data-sets have been generated for Breast-cancer^34^ and Ovarian Cancer^35^, LC-MS/MS raw files from COAD patients full-filled the required criteria and hence subjected to re-processing for lncRNA polypeptides identification. The COAD and normal colon LC-MS/MS data used in this publication was generated by the Clinical Proteomics Tumor Analysis Consortium (NCI/NIH). A total of 1220 mass-spectrometric (LC-MS/MS) raw files representing 92 colorectal adenocarcinoma (COAD) tissues and 30 normal colon epithelium tissues were analyzed for identification and quantification of lncRNA-peptidome (Supplementary Table S5).

**Figure 5:**
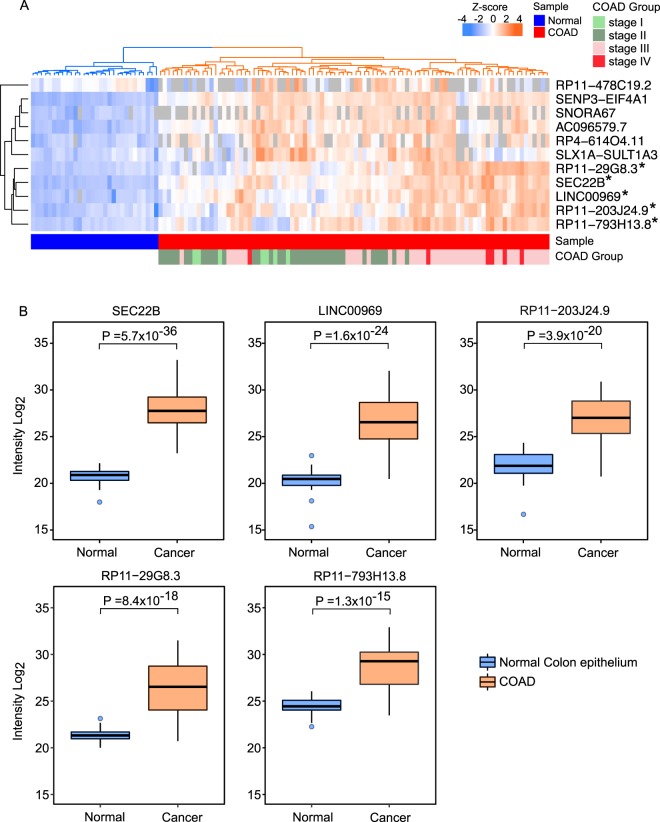
Higher abundance of UExp-polypeptides in COAD samples. (**A**) A heatmap representing the differential abundance of eleven lncRNA polypeptides across 92 COAD and 30 normal colon epithelial samples. The log_2_ transformed intensities (equivalent to relative abundance) of the polypeptides were z-scored before using in the heatmap. The color code gradient indicates the polypeptide intensity, where higher and lower intensities are marked by orange and blue color. Grey color indicated no abundance. The COAD and healthy samples were shown by the color coded horizontal bar bellow the heatmap where dark blue indicates normal colon samples whereas red indicates COAD samples. The hierarchical clustering algorithm was applied to both columns (samples) and rows (LncRNA polypeptide). Column (sample) clustering revealed two distinct clusters (indicated by color: blue and orange). UExp polypeptides were indicate by star (*). The different stages of COAD samples are shown by the color-coded horizontal bar bellow the heatmap (light and dark green indicates stage I and II respectively; light and dark red indicates stage III and IV respectively). (**B**) Box plot showing the average log_2_ transformed intensity of five lncRNA encoded ubiquitously expressed (UExp) polypeptides in normal colon epithelium (blue) and COAD tissues (orange). An unpaired t test was applied to identify the statistical significance of lncRNA polypeptide abundance variation between normal colon and COAD tissues. P values calculated by the unpaired t test are indicated.

In total 313 polypeptides, each encoded by a distinct lncRNA transcript, were identified in the COAD and normal colon tissues. Differential expression analysis revealed that eleven lncRNA polypeptides including five UExp-polypeptides were differentially abundant among the COAD and normal samples (Fig. 5A). A heatmap based on the z-scored log_2_ transformed intensities of these lncRNA polypeptides across 92 COAD and 30 normal colon tissues is shown (Fig. 5A). Hierarchical clustering of the samples (columns) and lncRNAs (rows) resulted in two distinct clusters separating the lncRNA polypeptide abundance-profile of normal and COAD samples. This clustering analysis was of particular importance because it revealed the power of differentially abundant lncRNA polypeptides to stratify the COAD and normal colon epithelium tissues separately. The higher abundance levels of the eleven lncRNAs were identified in the distinctly clustered COAD tissues compared to normal colon epithelium samples. Not all the lncRNA polypeptides were expressed uniformly in all the COAD and normal samples (the absence of quantification was represented by grey color in the heatmap, Fig. 5A). However, the five UExp-polypeptides (indicated by star in Fig. 5A) (SEC. 22B, RP11-29G8.3, LINC00969, RP11-203J24.9 and RP11-793H13.8) were expressed in all COAD and normal tissues. Next we wanted to know whether the abundance profiles of these five UExp lncRNA polypeptides were significantly higher in COAD compared to normal colon epithelium tissues. Indeed the five UExp lncRNA polypeptides were significantly upregulated in COAD in comparison to the normal colon tissues, justifying their candidacy as potential biomarkers for COAD (Fig. 5B). To gain mechanistic insight on the underlying reason behind the higher abundance of these five UExp-polypeptides in colon cancer compared to normal colon tissues, we hypothesized a transcription-driven model where the upregulation of lncRNA transcripts may lead to the higher rate of synthesis of these polypeptides. To test this, we searched the RNA-seq data from The Cancer Genome Atlas (TCGA) to analyze the lncRNA expression of the same COAD and normal colon tissue samples. Unfortunately only one lncRNA transcript (SEC. 22B) out of these five UExp lncRNAs was identified and more surprisingly it was not differentially regulated between COAD and normal colon tissues (data not shown). The absence of other lncRNA transcripts may well be due to their low expression levels in the COAD and normal colon samples. Failing to provide evidence for the transcription-driven model, we surmised a translational model where the upregulation of ribosome biogenesis and/or translational process in COAD samples may contribute to the higher rate of synthesis of these UExp-polypeptides. To verify this hypothesis, proteome data from COAD and normal colon samples were analyzed to identify the differentially regulated proteins (Fig. S4A) followed by gene-set enrichment analysis (Fig. S4B). The GO terms like epithelial-to-mesenchymal transition and MTORC1 signaling were highly enriched in COAD samples (Fig. S4B). Intriguingly, neither ribosome biogenesis nor any other ribosome/translation related pathways were upregulated in the COAD samples. Further investigations are required to elucidate the underlying mechanism of the upregulation of UExp lncRNAs in COAD samples.

**Figure 6:**
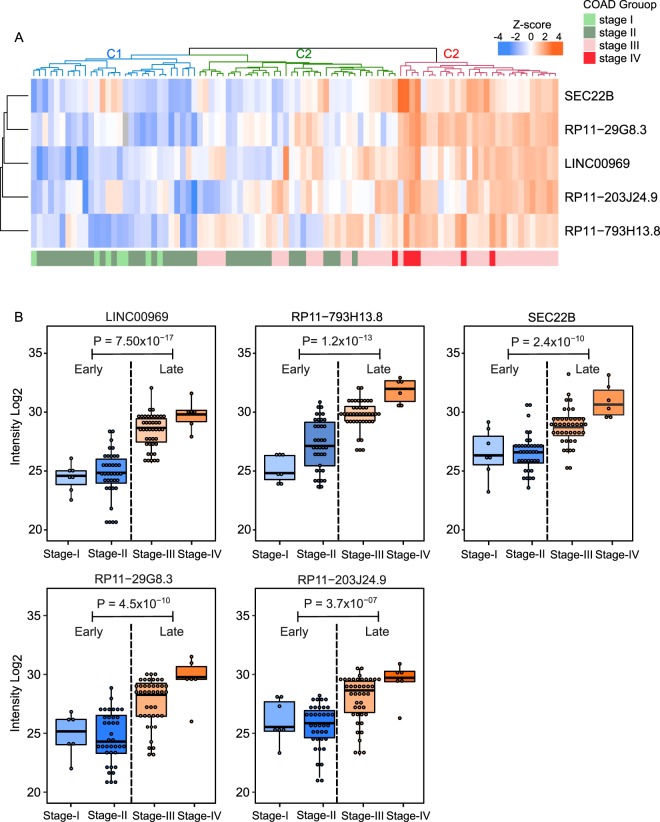
Abundance variation of UExp-polypeptides in different stages of COAD samples. (**A**) A heatmap signifying the abundance variation of five lncRNA encoded ubiquitously expressed (UExp) polypeptides across different stages (I, II, III and IV) of 92 COAD tissues. The log_2_ transformed intensities of the polypeptides were z-scored before using in the heatmap. The color code gradient indicates the polypeptide intensity, where higher and lower intensities are marked by orange and blue color. The different stages of COAD samples are shown by the color-coded horizontal bar bellow the heatmap (light and dark green indicate stage I and II respectively; light and dark red indicate stage III and IV respectively). The hierarchical clustering algorithm was applied to both columns (samples) and rows (LncRNA polypeptide). Column (sample) clustering revealed three distinct clusters denoted as C1, C2 and C3. (**B**) Box plot showing the average log_2_ transformed intensity of five lncRNA encoded ubiquitously expressed (UExp) polypeptides in different stages of COAD tissues. Due to uneven number of samples among the four stages of COAD, the total samples were divided into early (Stage I and II) and late (stage III and IV) stages for statistical analysis. An unpaired t test was applied to identify the statistical significance of lncRNA polypeptide abundance variation between early and late stages COAD tissues. P values calculated by the unpaired t test are indicated.

Higher variability of the UExp polypeptide abundance-profiles within the COAD patients compared to that of normal individuals was evident (Fig. 5B). Whether the observed higher variation in the COAD group is an artifact due to the large number of samples (n = 92) compared to normal colon epithelium group (n = 30) or may originate from some biological factors such as cancer heterogeneity was unclear. Since cancer tissue samples can vary greatly depending on the clinical features of the cancer, we reasoned that the different clinical staging of the COAD samples may attribute to the high variability of the UExp polypeptides abundance-profiles. To investigate this, we analyzed the distribution of the UExp-polypeptides abundance profile across different clinical stages of CAOD. Intriguingly hierarchical clustering of the COAD samples (columns) showed three distinct clusters (C1, C2 and C3) (Fig. 6A). The COAD samples representing early-stages (Stage I and II) were grouped together representing one distinct cluster (Fig. 6A). On the other hand, the late-stage (Stage III and IV) COAD samples were clustered together distinctly from the early-stage samples. The abundances of the UExp-polypeptides were higher in the late-stage (Stage III and IV) compared to early-stage COAD samples. Interestingly another cluster (middle) representing the mixed stages (Stage II and III) of COAD samples showed intermediate levels of abundance profile of lncRNA polypeptides. All together stage-wise clustering of COAD tissues revealed the definite correlation of the UExp-polypeptides abundance with the clinical staging of COAD samples. Further analysis revealed that a gradual increase in the abundance of UExp-polypeptides from stage I to stage IV (Fig. 6B). To test whether the clinical-stage dependent increase of abundance of UExp-polypeptides are statistically significant, ANOVA test was employed. Due to the unequal number of patients of different stages, we divided the stages into early (combining the patients of Stage I and II) and late (combining the patients of Stage III and IV). Significant higher abundance of UExp-polypeptides was observed in case of the late-stage COAD samples (Stage III + IV) compared to the samples of early-stage (Stage I + II), indicating that the expression of these lncRNA polypeptides were somehow related to the cancer progression (Fig. 6B).

In summary, the results unveiled a distinct molecular signature of the COAD tissues based on the differential abundance profile of lncRNA-peptidome. The lncRNA-peptidome signature apparently harbors the capacity to distinguish the cancer from healthy tissues as well as to differentiate the stages of colon cancer and thus strengthens the promise as potential prognostic marker.

### Potential of lncRNA polypeptides as tissue and plasma-based diagnostic marker for prostate cancer

**Figure 7:**
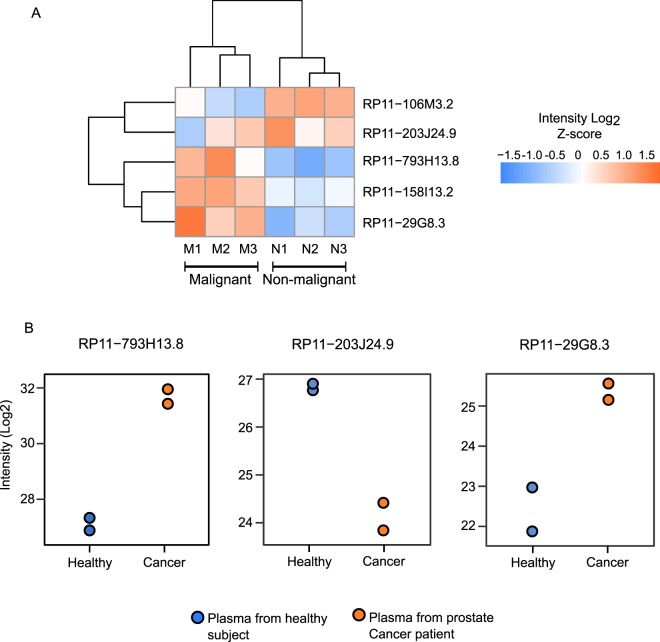
Differential abundance of UExp-polypeptides in tissue and plasma samples of prostate cancer patients. (**A**) Heatmap representing the abundance profile of five lncRNA polypeptides across three patient-matched malignant and adjacent non-malignant tissues from prostate cancer patients. The columns (malignant/non-malignant tissues) and rows (LncRNA polypeptides) were clustered by using hierarchical clustering algorithm. The color code represents the intensities of the polypeptides where orange and blue indicate high and low intensities respectively. UExp polypeptides were indicate by star (*). (**B**) Abundance plot showing the log_2_ transformed intensity of three ubiquitously expressed (UExp) lncRNA encoded ubiquitously expressed (UExp) polypeptides in the plasma samples from healthy and prostate cancer patients.

Inspired by the substantial ability of the lncRNA polypeptides to stratify COAD patients from the normal individuals, we investigated whether their potential as a biomarker is restricted to COAD or we can harness lncRNA-peptidome for diagnosis of other cancers as well. With that view we selected prostate cancer for which LC-MS/MS raw data were available for tissues and plasma samples. To investigate the molecular signature of lncRNA-peptidome in prostate cancer tissue we re-processed the LC-MS/MS raw files from three patient-matched malignant and adjacent non-malignant prostate tissues. In total five common lncRNA polypeptides were quantified among malignant and adjacent non-malignant tissues. Out of the five common polypeptides three were UExp-polypeptides (RP11-793H13.8, RP11-29G8.3 and RP11-203J24.9). Clustering analysis based on the z-scored abundance of these lncRNA polypeptides revealed two distinct clusters (Fig. 7A). One cluster signified malignant and another represented non-malignant tissue corroborating the hypothesis that lncRNA-polypeptides has the ability to stratify malignant cancer tissues from their normal counterparts. Out of these lncRNA-polypeptides, three (RP11-793H13.8, RP11-158I13.2 and RP11-29G8.3) showed higher abundance in malignant tissues compared to non-malignant ones (Fig. 7A). Intriguingly, two remaining lncRNA polypeptides (RP11-106M3.2 and RP11-203J24.9) displayed the opposite trend where their abundance is lower in malignant tissues compared to the non-malignant prostate tissues (Fig. 7A). Differential abundance analysis revealed that three UExp (RP11-793H13.8, RP11-158I13.2, RP11-29G8.3) and one non-UExp (RP11-106M3.2) polypeptides were significantly different between malignant and non-malignant tissues (Supplementary Table S6).

Since plasma is a readily accessible biological sample collected from patients, plasma proteomics-analysis would be ideal for cancer diagnosis. To investigate whether these lncRNA polypeptides can be quantified in the plasma samples and importantly maintain their differential abundance pattern between healthy and prostate cancer patients, we re-processed the LC-MS/MS raw files representing plasma proteome of two healthy and two prostate cancer patients. In total, ten lncRNA polypeptides were identified as overlapping between the plasma samples from healthy and prostate cancer subjects (Figs 7B and S5). Out of these, three UExp lncRNA polypeptides showed significant abundance deviation between prostate cancer and normal plasma samples. Interestingly a marked increase in the abundance of RP11-793H13.8 and RP11-29G8.3 polypeptides in the plasma of prostate cancer patients was observed (Fig. 7B). Opposite to this trend, RP11-203J24.9 polypeptide showed noticeable reduced abundance in the plasma samples of prostate cancer patients compared to healthy samples. To compare the abundance rank of these three plasma lncRNA polypeptides with that of FDA approved plasma-biomarkers, LC-MS/MS raw files representing the plasma proteome of healthy individuals were retrieved from a study conducted by Geyer *et al*.^36^. After re-processing of the raw files by MaxQuant, a standard abundance profile of the plasma proteome was established. To facilitate the comparison, the abundance of the candidate lncRNA-polypeptides (RP11-793H13.8, RP11-29G8.3 and RP11-203J24.9) from prostate cancer patients and healthy individuals were superimposed on standard plasma abundance profile. The three lncRNA polypeptides showed an intermediate abundance rank (Fig. S6). This result suggested the feasibility of the detection of these polypeptides with conventional immunological assays as their dynamic range falls within the detectability with that of FDA approved plasma biomarkers.

## Discussion

Low abundance of lncRNA transcripts in plasma samples, attributed by their unstable nature has challenged the candidacy of circulating lncRNAs as potential biomarker in recent times^23^. In the current study, we proposed the translational product of lncRNAs – designated as lncRNA-polypeptides as potential biomarkers for different cancer types which may overcome the inherent stability issue that are associated with RNA molecules. The lncRNA-polypeptides have obvious advantages over their lncRNA encoders. For instance polypeptides/proteins are shown to be five times more stable and 2800 time more abundant than mRNAs in mammalian systems^32^. Moreover the proposed lncRNA polypeptides based biomarkers are not limited by cancer types rather was shown to be applicable for different cancers such as COAD and prostate cancer.

We took advantage of an in-house computational-proteogenomic workflow that includes the genome-informed identification of lncRNA-polypeptides by reprocessing LC-MS/MS raw data. By employing this workflow we analyzed the lncRNA-peptidome in 14 human tissues and 11 cell lines, which led to the identification of numerous tissue-specific as well as five universally expressed (UExp) lncRNA polypeptides. It has been proposed that the translational efficiency of cytoplasmic lncRNAs is comparable to that of mRNAs^37^. As an extension of this hypothesis and to provide proteome-centric evidence, the current study also revealed the comparable abundance of lncRNA-peptidome and proteome implying the analogous translational efficiency of lncRNAs and mRNAs in human tissues.

Intriguingly an opposing scenario emerged when proteomes from tissues and cell lines were compared to the respective lncRNA-peptidomes. A uniform abundance profile of proteomes and lncRNA-peptidomes were evident in cell lines whereas tissue proteomes and their respective lncRNA-peptidomes exhibited more variability across different tissues. Previously, this unanticipated high degree of uniformity of the proteomes of the different cell lines was assumed as the consequence of the adaptation of cell lines to the indefinite growth under *in-vitro* conditions^33^. In light of this argument, we surmise that the high consistency of the cell line lnRNA-peptidomes presumably originates partly from the fact that cell lines represents *in-vitro* culture of a single cell type devoid of the tissue-microenvironment. On the contrary, primary tissues are typically composed by multiple cell types and maintain a constant communication with the microenvironment. Another striking difference between the primary tissues and cell lines is the pattern of correlation between proteomes and lncRNA-peptidomes. Comparison between the abundance profiles of lncRNA-peptidomes and proteomes showed a positive correlation across tissues while an inverse correlation was observed across cell lines. This opposing correlation trend posed an interesting scenario, which requires further investigations. This inconsistency is likely to be originated from biological difference between tissues and cell line rather than technical biasness. The abundances of the lncRNA polypeptides and proteins in the tissues and cell lines were normalized by using MaxLFQ algorithm^38^, which can overcome the problem of comparing different samples such as cell lines and tissues that may have been processed in different ways^38^. Moreover, to correct for technical bias that may exist between tissue and cell line datasets, the number of lncRNA polypeptides were adjusted with respect to the total number of proteins identified in matched tissues and cell line samples. Subsequently, lncRNA-peptidome was expressed as fraction of total proteome. The concordant correlation of the lncRNA-peptidome fraction of the total proteome between tissue-cell line pairs further boosted our confidence that the possibility of technical bias is minimal, hence, strengthen the assumption that the observed difference regarding the lncRNA-peptidome and proteome correlation between cell line and tissues is like to be biological in nature. In our study, the result suggests that in primary tissues, the abundance of lncRNA-peptidome changes as a function of proteome abundance. Earlier it has been proposed that the translational rate is the major contributor for the variation in protein abundance in mammalian cells^37^. Moreover tissue-specific differences in the rate of protein synthesis have previously been shown for different tissues, which are assumed to play a major role in the regulation of tissue homeostasis^39^. In line with this hypothesis, we assumed that the positively correlated global abundance of lncRNA-peptidomes and proteomes across different tissue may have emerged from the tissue-specific translational capacity of the ribosomal machineries that do not discriminate mRNAs from lncRNA transcripts. On the contrary, the negative correlation between the lncRNA-peptidome and proteome abundance profiles in the cell lines may likely reflect biological difference between tissues and cell-line that requires further investigation.

Regardless of these differences between primary tissues and cell lines, we identified five UExp lncRNA polypeptides that showed universal expression across the analyzed tissues and cell lines. By comparing lncRNA-peptidome of cancer tissues (colon and prostate) and their normal counterparts, we were able to unearth the potential of these five UExp lncRNA-polypeptides (RP11-793H13.8, RP11-203J24.9, SEC. 22B, LINC00969, RP11-29G8.3) as candidate biomarkers for these cancers. The five UExp-polypeptides were quantified in each of the normal colon (n = 30) and COAD tissue (n = 92) samples whereas the rest of the identified lncRNAs polypeptides were not quantified in all the analyzed tissue samples. This result suggested that the five UExp-polypeptides were pervasively translated in all the colon tissues and not affected by the genomic variability of individuals. The gradual stage-dependent increment in the abundance of these UExp-polypeptides in colon adenocarcinoma (COAD) underlines their potential as prognostic biomarkers in COAD. The significant abundance deviation of these UExp-polypeptides between late and early stages COAD strengthens their candidacy as prognostic-biomarkers. The underlying reason behind the upregulation of the UExp-polypeptides remains unclear. To uncover any causal relationship that may exists between the upregulation of UExp-polypeptides and progression of COAD requires in-depth understanding of the regulation of lncRNA translational process. With the aim to gain mechanistic insights into the regulation of UExp-polypeptides synthesis we investigated the plausible transcriptional and translational regulations. However, the results were not sufficient to identify any specific mechanism and further detailed analyses are required to validate the transcriptional and/or translational mechanisms that may underlie the higher abundance of the UExp-polypeptides in COAD tissues. Nevertheless, it has not escaped out attention that a third possible mechanism involving the increased stability and/or reduced degradation of the UExp-polypeptides may exist, that may explain their higher abundance. Previously it was shown that downregulation of proteolytic systems may lead to the decreased degradation rates of onco-proteins in human cancer^40^. Although it is plausible that analogous mechanism may reduce the degradation of the UExp-polypeptides in COAD tissues, further studies are needed to prove this concept. Moreover, it has been proposed that proteomic instability is inherently associated with cancer and is considered to have tumor suppressor effect^41^. To avert this intrinsic tumor-suppressor activity of proteomic instability, many type of cancer cells utilize constitutive activation of HSF1 to stabilize and maintain proteome homeostasis^41^. Whether a similar mechanism contributes to the higher stability of the lncRNA-peptidome in cancer cells remains to be elucidated.

Having analyzed the UExp-polypeptides in COAD, we investigated whether the potential of these UExp-polypeptides is only restricted to COAD or can be extend to other cancer types. By comparing the lncRNA-peptidome of prostate-cancer tissues and adjacent cancer-free tissues, we showed that the UExp-polypeptides to be differentially regulated in prostate cancer tissues. Unlike the COAD, not all the UExp-polypepitdes in prostate cancer tissues were upregulated. In contrary to the upregulated UExp-polypepitdes (RP11-793H13.8 and RP11-29G8.3), one UExp-polypeptide RP11-203J24.9 was downregulated in malignant prostate-tissues compared to the non-malignant counterparts (Fig. 7A). This result signifies that although the differential expression of lncRNA polypeptides can be observed in different cancer types, the up- or down-regulation seems to be cancer specific, highlighting a cancer-specific regulation of lncRNA polypeptides. Next we asked, whether the differential abundance of UExp-polypepitdes can also retained in the plasma samples. Analysis of the plasma samples showed that the differential abundance of the UExp-polypeptides that was observed in the prostate tissues was also reflected in the plasma samples of prostate cancer patients. Importantly the tissue-specific up-regulation of the two UExp-polypepitdes (RP11-793H13.8 and RP11-29G8.3) and down-regulation of one UExp-polypeptide (RP11-203J24.9) were mirrored in plasma samples of prostate cancer patients as well. The possible circulatory nature of the lncRNA polypeptides was strengthened by the presence of three lncRNA encoded polypeptides (RP11-793H13.8 and RP11-29G8.3 and RP11-203J24.9) in the plasma samples. The lncRNA-polypeptides were shown to occupy an intermediate abundance rank when compared to the plasma proteome abundance including FDA approved biomarkers (Fig. S6). This result hints towards a possibility of quantification of these polypeptides using typical immunological assays.

An interesting aspect of the proposed lncRNA derived UExp-polypeptides is their universal expression, which indicate towards their potency as common biomarkers for various cancer types with diverse tissue origin. The scarcity of effective biomarkers with substantial prognostic power is posing a great challenge to manage and treat cancer patients. Harnessing the LncRNA-peptidome in tissue and plasma samples from cancer patients may open a window of opportunity to explore novel biomarkers for cancer and consequently reshape the future in cancer diagnosis and prognosis.

## Materials and Methods

### Generation of custom build lncRNA polypeptide database by three-frame translation of the lncRNA transcripts

To generate the hypothetical lncRNA polypeptide database, first the nucleotide sequences of long non-coding RNA (lncRNA) transcripts were retrieved from GENCODE V30 (GRCh37.p13). In total 23,898 lncRNA transcripts on the reference chromosomes were obtained. Here we adopt a proteo-genomic workflow that is typically based on the mapping of tandem MS/MS spectra derived from the peptides onto the hypothetical protein/polypeptide database via three-frame translation of the lncRNA transcripts. To this end the lncRNA transcripts were subjected to *in-silico* three-frame translation to obtain the hypothetical polypeptide sequences encoded by lncRNA transcripts. Additionally the annotations of lncRNA transcripts were further validated by LNCipedia (Version 5.2)^42^.

### Retrieving mass-spectrometry raw files from PRoteomics IDEntifications (PRIDE) database

PRoteomics IDEntifications (PRIDE) database containing the LC-MS/MS raw files were systematically mined to retrieve 1346 of raw files from diverse array of human samples including tissues, cell lines, cancer tissues and plasma (Supplementary file S1). For human tissue mass-spectrometry raw files were obtained from Project PXD000561 deposited in PRIDE database by Kim *et al*.^43^. In total 972 number of raw files representing 14 different human tissues (Heart, Frontal cortex, Urinary Bladder, Testis, Prostate, Liver, Adrenal gland, Ovary, Spinal Cord, Colon, Lung, Pancreas and Kidney) were retrieved. The raw files were generated by LTQ Orbitrap Elite and LTQ Orbitrap Velos instrument. For sample processing the extracted protein samples were first separated by SDS-PAGE and in-gel digestion was carried out using trypsin. For human cell lines the raw files were taken from Project PXD002395 deposited in PRIDE database by Geiger *et al*.^33^. In total 198 of raw files representing the proteome of 11 different cell lines - A549, GAMG, HEK293, HeLa, HepG2, K562, MCF7, RKO, U2OS, LnCap and Jurkat were obtained. For Colon cancer (colon adenocarcinoma: COAD) we took the advantage of the huge number of LC-MS/MS raw file (n = 576) representing colon tissues from 92 COAD patients under the PRIDE project: PXD002080. The PRIDE project PXD00208 was deposited by Zhang *et al*. as a part of Clinical Proteomic Tumor Analysis Consortium (CPTAC) involving proteomics analysis of clinically characterized colorectal adenocarcinoma (COAD) samples^44^. The clinical features and metadata for the 92 COAD tissue samples generating these 920 raw files are given in Supplementary File 2. For normal colon epithelium analysis, 300 LC-MS/MS raw files were retrieved from CPTAC (https://cptac-data-portal.georgetown.edu/cptac/s/S019). For prostate cancer, 36 raw files representing six prostate cancer tissues were retrieved from the PRIDE database under the project IDs: PXD004132^45^. Lastly 4 raw files from PRIDE database (PXD001194) representing 4 plasma samples from healthy (n = 2) and prostate cancer (n = 2) subjects were obtained^46^.

### Identification and quantification of lncRNA polypeptides by MaxQuant

Individual LC-MS/MS raw files retrieved PRIDE database representing mass-spectrometry measurements of various human healthy and cancer tissues, cell lines and plasma samples were analyzed by MaxQuant^47^ (version: 1.6.0.1). All the MaxQuant parameters were set as described previously by Wilhelm *et al*.^28^. Briefly, the MS/MS spectra were searched by Andromeda search engine^48^ implemented in Maxquant against the custom-built merged FASTA database encompassing all the peptide sequences from hypothetical human lncRNA-peptidome and proteome. MaxQuant analysis included an initial search with a precursor mass tolerance of 20 ppm, the results of which were used for mass recalibration. In the main Andromeda search precursor mass and fragment mass had an initial mass tolerance of 6 ppm and 20 ppm, respectively. The search included variable modifications of methionine and oxidation, and N-terminal acetylation, and fixed modification of carbamidomethyl cysteine. Minimal peptide length was set to six amino acids and a maximum of two missed-cleavages were allowed. The false discovery rate was set to 0.01 for peptide and protein identifications. In the case of identified peptides that are all shared between two lncRNA, these peptides are combined and reported as one lncRNA polypeptides. For quantification purpose MaxLFQ algorithm^38^ was employed for the quantification purpose. To increase the reliability and reduce ambiguity in the identification of the lncRNA encoded peptides stringent criteria was set. For instance the minimum number of non-overlapping unique peptides required to report an identification/quantification of corresponding polypeptide was set as two. In all the analyzed LC-MS/MS raw files, the human tissue and plasma samples were predigested with Trypsin enzyme, hence only trypsinized peptides (with c-terminal Arg or Lys) were identified by MaxQuant by the default setting of MaxQuant where trypsinized peptides were preferentially identified. Additionally the possibility of sequence-matching between the lncRNA translational product and known human proteins has been nullified by performing BLAST of the predicted lncRNA peptides against the Uniprot human proteome database (https://www.uniprot.org/proteomes/UP000005640). The lncRNA peptides that matched with human proteins were discarded and only the peptides that exhibited no-match to human proteins were considered for further analysis.

### Differential-expression analysis

To assess statistical significance of lncRNA regulation between cancer samples, healthy tissues and cell lines, we used a linear model-based approach (Limma R package^49^). Briefly the colon cancer (COAD) samples were compared to healthy colon tissues. Similarly malignant and non-malignant prostate samples were compared to identify the differentially abundant lncRNA polypeptides. Adjusted p-value bellow 0.05 was set as significant threshold. One-way ANOVA was used to assess the significance of lncRNA different abundance across cancer stages, i.e Stage I to IV in TCGA COAD samples. Briefly we first selected lncRNAs that have been quantified in at least 2 out of 4 samples in each cancer stage. For each of the lncRNAs that passed this threshold, we applied the one-way ANOVA to compare mean abundance in stage I to IV. For prostate cancer we applied the same method comparing abundance of the 10 lncRNAs quantified in the two healthy and two cancer samples.

### Gene-set enrichment analysis

Enriched biological processes from Gene Ontology database were retrieved using the list of significantly regulated protein between COAD and healthy colon (adj. pvalue < 0.05) in a hypergeometric test compared to the whole set of quantified. Significance threshold was set to adjusted p-value bellow 0.05.

## Supplementary information

Supplementary Figure 1 to 7

Table S1

Table S2

Table S3

Table S4

Table S5

Table S6

## Data Availability

This study is based on freely accessible datasets listed in Table S1.
