# Supplementary material for: Harnessing the tissue and plasma lncRNA-peptidome to discover peptide-based cancer biomarkers
Source: Sci Rep. 2019 Aug 23;9:12322. doi: 10.1038/s41598-019-48774-1 (PMC6707329; doi:10.1038/s41598-019-48774-1)

Title: Harnessing the tissue and plasma lncRNA-peptidome to discover peptide-based cancer biomarkers

Sajib Chakraborty[1#], Geoffroy Andrieux[2,3,4#], A M Mahmudul Hasan[1], Musaddeque Ahmed[5], Md. Ismail Hosen[1], Tania Rahman[1], M. Anwar Hossain[1] and Melanie Boerries [2,3,4,6*]

[1] Molecular systems biology laboratory, Department of Biochemistry and Molecular Biology, University of Dhaka, Bangladesh

[2] Institute of Medical Bioinformatics and Systems Medicine, Medical Center - University of Freiburg, Faculty of Medicine, University of Freiburg, Freiburg, Germany

[3] Institute of Molecular Medicine and Cell Research, Faculty of Medicine, University of Freiburg, Freiburg, Germany

[4] German Cancer Consortium (DKTK) and German Cancer Research Center (DKFZ), Partner Site Freiburg, Freiburg, Germany

[5] Princess Margaret Cancer Centre/University Health Network, Toronto, Ontario, Canada

[6] Comprehensive Cancer Center Freiburg (CCCF), Medical Center – University of Freiburg, Faculty of Medicine, University of Freiburg, Germany

# authors contributed equally

*corresponding author: Prof. Dr. Dr. Melanie Boerries, Institute of Medical Bioinformatics and Systems Medicine, Medical Center – University of Freiburg, Faculty of Medicine, University of Freiburg, Freiburg, Germany

E. mail: m.boerries@dkfz-heidelberg.de

Telephone: +49 761 270-84671

Legend of Supplementary Tables

Table S1: Description of datasets

Table S2: Detailed annotations of lncRNA transcripts with coding potential in human tissues

Table S3: Detailed annotations of lncRNA transcripts with coding potential in cell-lines

Table S4: Sequences and LFQ quantification of peptides from the five UExp-polypeptides

Table S5: Summary of analysed COAD samples

Table S6: Malignant vs. Non-malignant differential analysis of lncRNA polypeptides

Legend of Supplementary Figures

Figure S1: LncRNA peptides, polypeptides and proteome abundance profile in tissues

The bar diagrams representing the abundance profiles of peptides encoded by LINC00969 (A) and RP11-203J24.9T (B) are shown across tissues. Each bar indicates the mean abundance (log_2 transformed intensity) of the peptides identified as a translational product of a given lncRNA. The colored circles represent individual peptides encoded by the lncRNA. The number at the base of the bars denotes the coefficient of variation (%CV). ANOVA test was carried out to test whether the mean abundances of the peptides were significantly deviating across tissues. P value indicates the level of significance.

(C) The global abundance profile comparison of tissue-specific proteome and lncRNA-peptidome. The bar diagram represents global abundance profile of the proteins and lncRNA polypeptides that were identified in each tissue. Each bar indicates the average intensity (Log_2) of all identified proteins (Blue) and lncRNA polypeptides (Orange) in a particular tissue. The error bar denotes the standard error of mean (SEM) of the log transformed intensities.

Figure S2: LncRNA polypeptides abundance profile in cell lines and identification in tissue-cell line pair

(A) The global abundance profile comparison of cell line-specific proteome and lncRNA-peptidome. The bar diagram represents global abundance profile of the proteins and

lncRNA polypeptides that were identified in each cell line. Each bar indicates the average intensity (Log_2) of all identified proteins (Blue) and lncRNA polypeptides (Orange) in a particular cell line. The error bar denotes the standard error of mean (SEM) of the log transformed intensities.

Percentage of lncRNA-peptidome overlap for Colon-RKO (B), Prostate-LnCAP (C), Frontal Cortex-GAMG (D), Liver-HEPG2 (E) and Lung-A549 (F) are shown. The Y-axis represents the number of lncRNA polypeptides identified. The black circles indicate the source of the lnRNA polypeptides (tissue and cell line). The single black circle indicates the number of lncRNA polypeptides (vertical bar) that were exclusively identified either in a given tissue or a cell line whereas the connected circles indicates the number of lncRNA polypeptides that overlap between a tissue-cell line pair. The horizontal bars represent the total number of lncRNA polypeptides identified in a particular tissue and cell-line.

(G) LncRNA-peptidome was expressed as a fraction of the total proteome by adjusting the number of lncRNA polypeptides with the total number of proteins in particular tissue and cell-line samples. LncRNA-peptidomes as a fraction of total proteomes are shown in the correlation plots representing five matched tissue-cell line pairs (Frontal Cortex-GAMG, Liver-HepG2, Prostate-LnCAP, Lung-A549 and Colon-RKO). The Pearson correlation coefficient (R) is shown on bottom right corner for each plot. P-value shows the significance of the correlation plots as calculated by t-test.

Figure S3: MS/MS spectra for four lncRNA encoded peptides

MS/MS spectra for the peptides – KHTLSYVDGTGK (A), ESTGAQVQVAGDMLPNSTER (B), THETSAHEGQTEAPSIDEK (C) AND VNAEGSVDSVFSQVCTHLDFLK (D) representing LINC00969, RP11-793H13.8, RP11-29G8.3, RP11-203J24.9 respectively are shown. MaxQuant viewer option was utilized to visualize the spectra.

Figure S4: Differential expression analysis (DEA) between the proteomes of COAD and normal colon epithelium tissues

(A) Scatter plot showing the differential expression of proteins in COAD samples in comparison to normal colon epithelium tissues. X-axis represent the average Log_2 protein fold change between COAD (n=92) and normal colon epithelium (n=30) tissues. Y-axis represents the combined intensity of each protein in COAD and normal colon tissues. Each dot (data point) represents a single protein. Proteins are colored according to their P-value as calculated by Limma analysis. Orange and blue colored dots represent the upregulated and downregulated proteins, respectively in COAD tissues.

(B) GO enrichment analysis on the proteins that are significant differentially regulated in across COAD and normal colon tissues. Enrichment scores for the upregulated pathways (orange bars) in COAD tissues are shown as -log10 p-values for each biological process whereas downregulated pathways (blue bars) in COAD tissues are shown as log10 p-values. P-values ($-log_{10}$), lower than 0.05 are shown on the x-axis.

Figure S5: Abundance profile of lncRNA polypeptides in serum samples from healthy and prostate cancer patients

Abundance plot showing the log_2 transformed intensity of seven lncRNA encoded polypeptides in the plasma samples from healthy individuals (n=2) and prostate cancer patients (n=2).

Figure S6: Distribution of proteins and lncRNA encoded polypeptides quantified in the human plasma samples

Distribution of proteins quantified in the human plasma as reported by Geyer *et al.* [36]. Three FDA-approved biomarkers are color coded (yellow). The biomarkers are chosen based on their abundance rank. APOC3, CRP and PSMA5 represent high, intermediate and low abundance profiles. Abundance rank of three lncRNA encoded polypeptide biomarkers in the plasma samples of healthy (A) and prostate cancer patients (B) are superimposed on the abundance rank profile of plasma proteome. Three lncRNA encoded polypeptide biomarkers are shown as orange circles.

Supplementary Figure S1

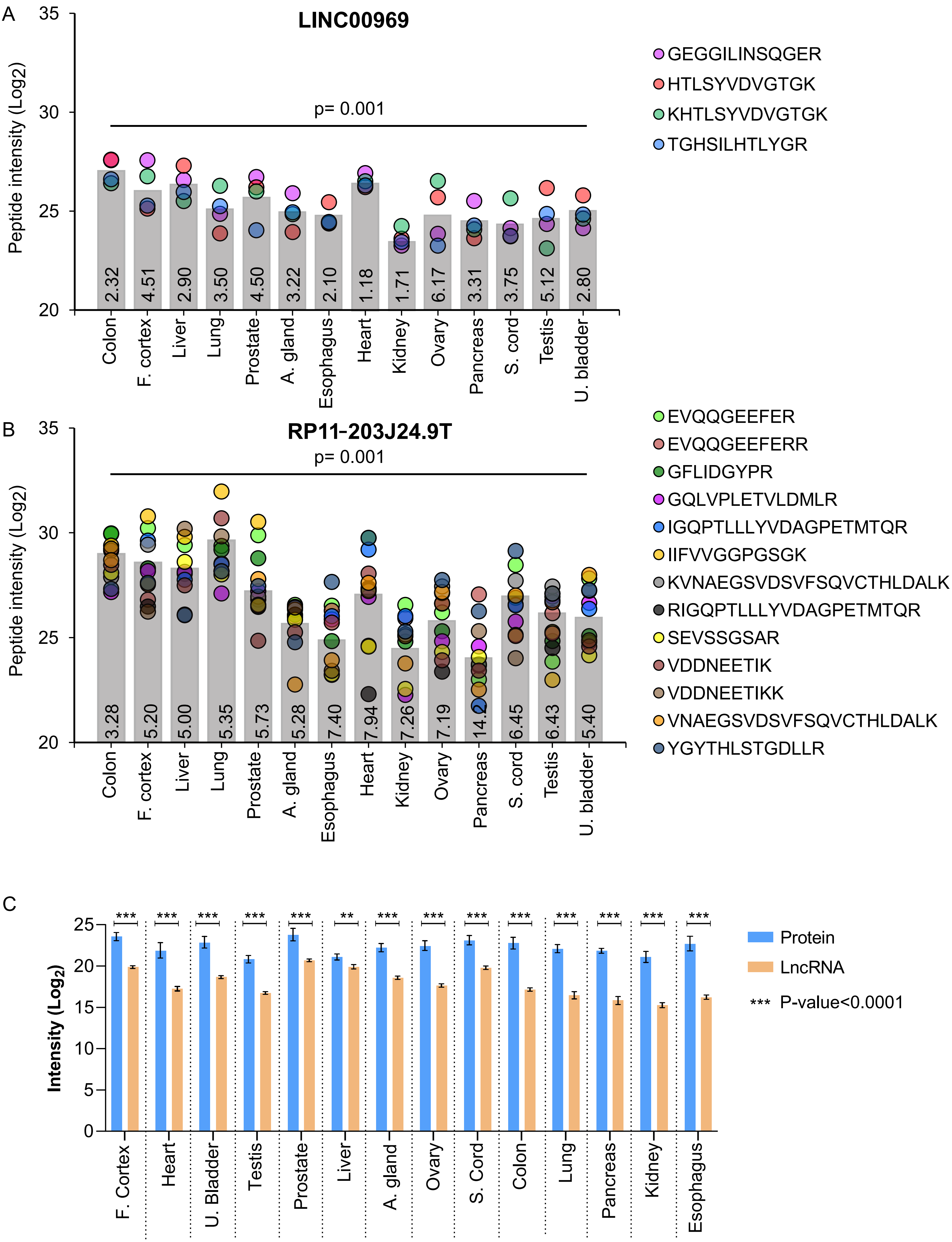

Supplementary Figure S2

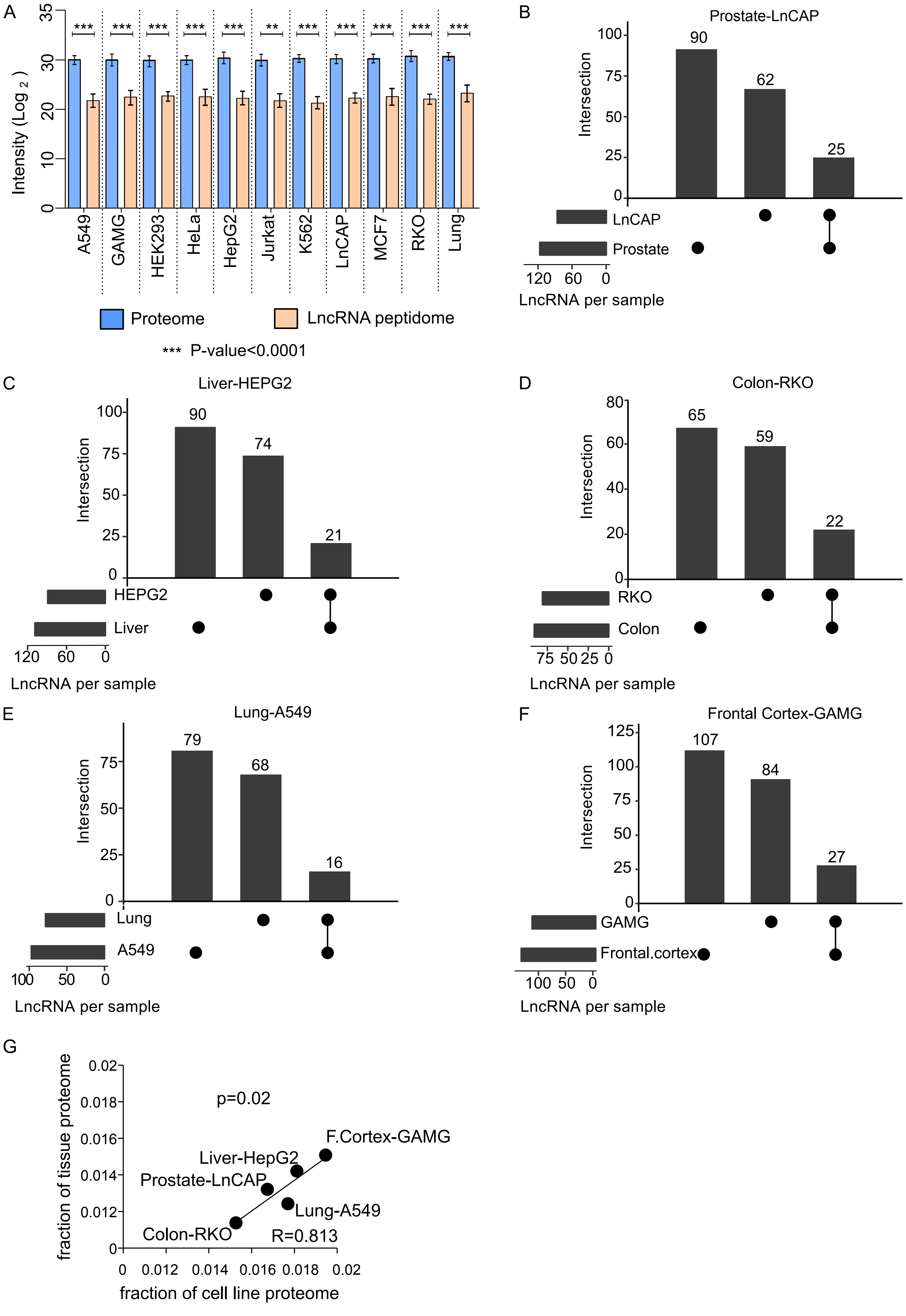

Supplementary Figure S3

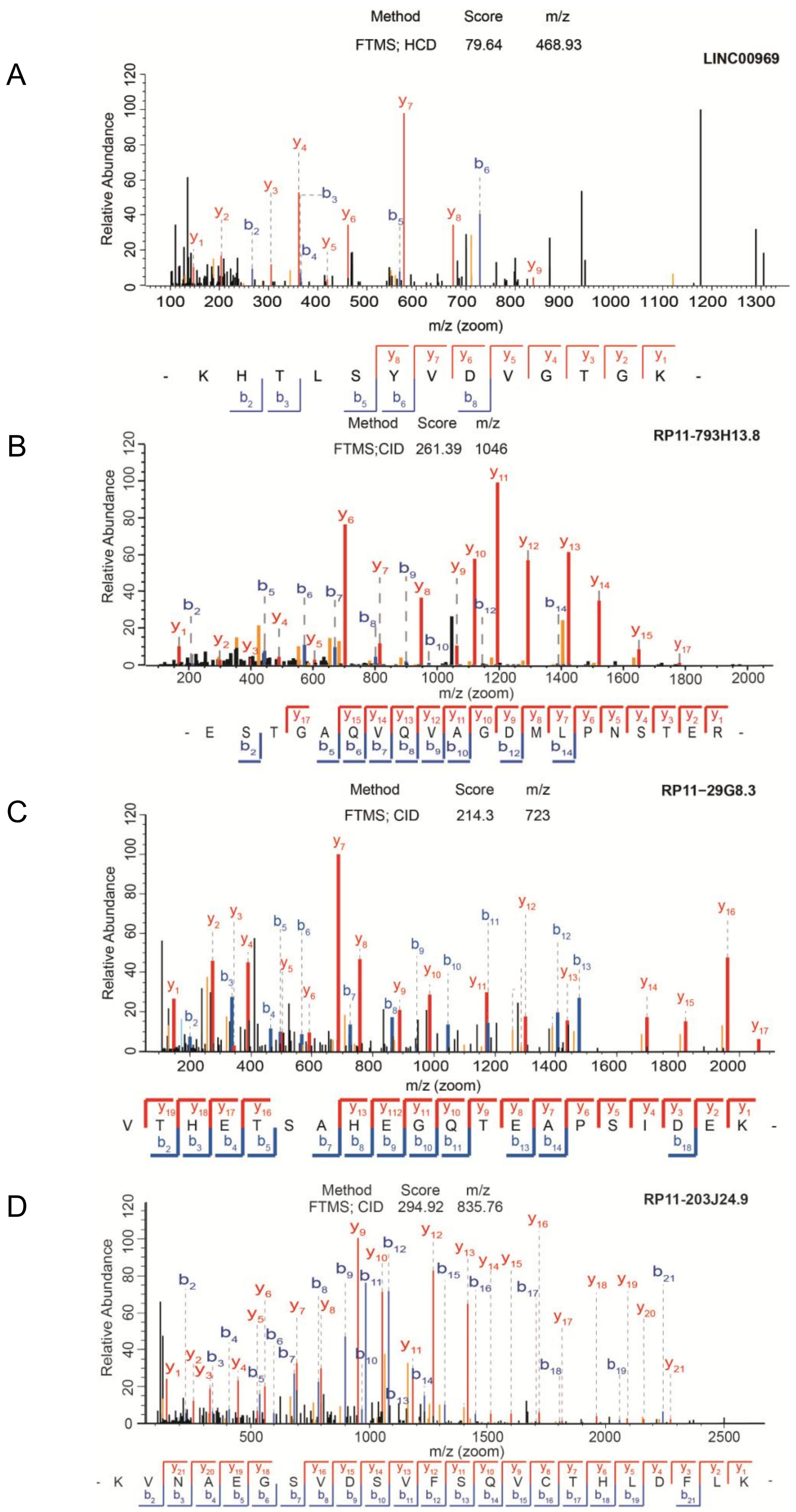

Supplementary Figure S4

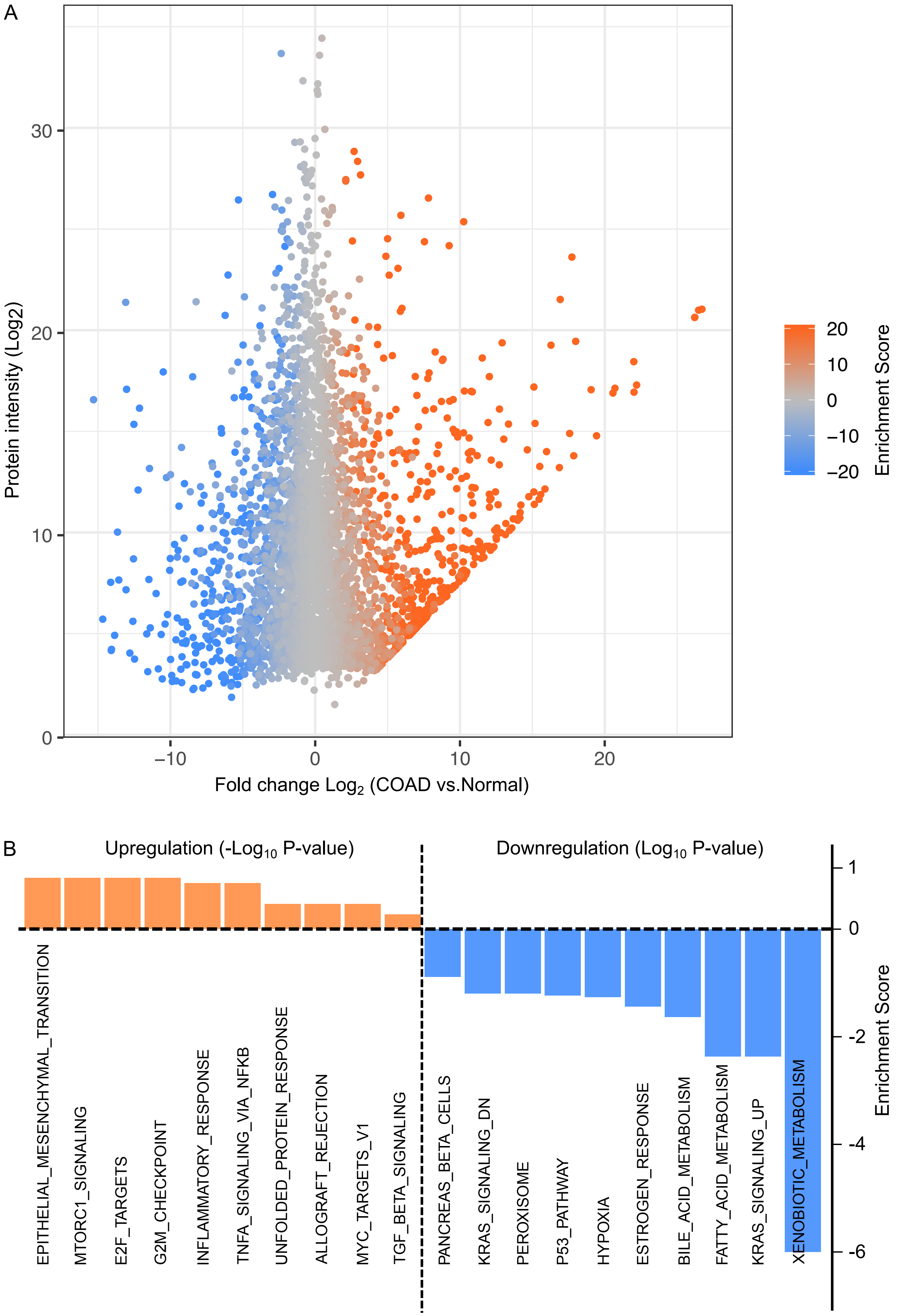

Supplementary Figure S5

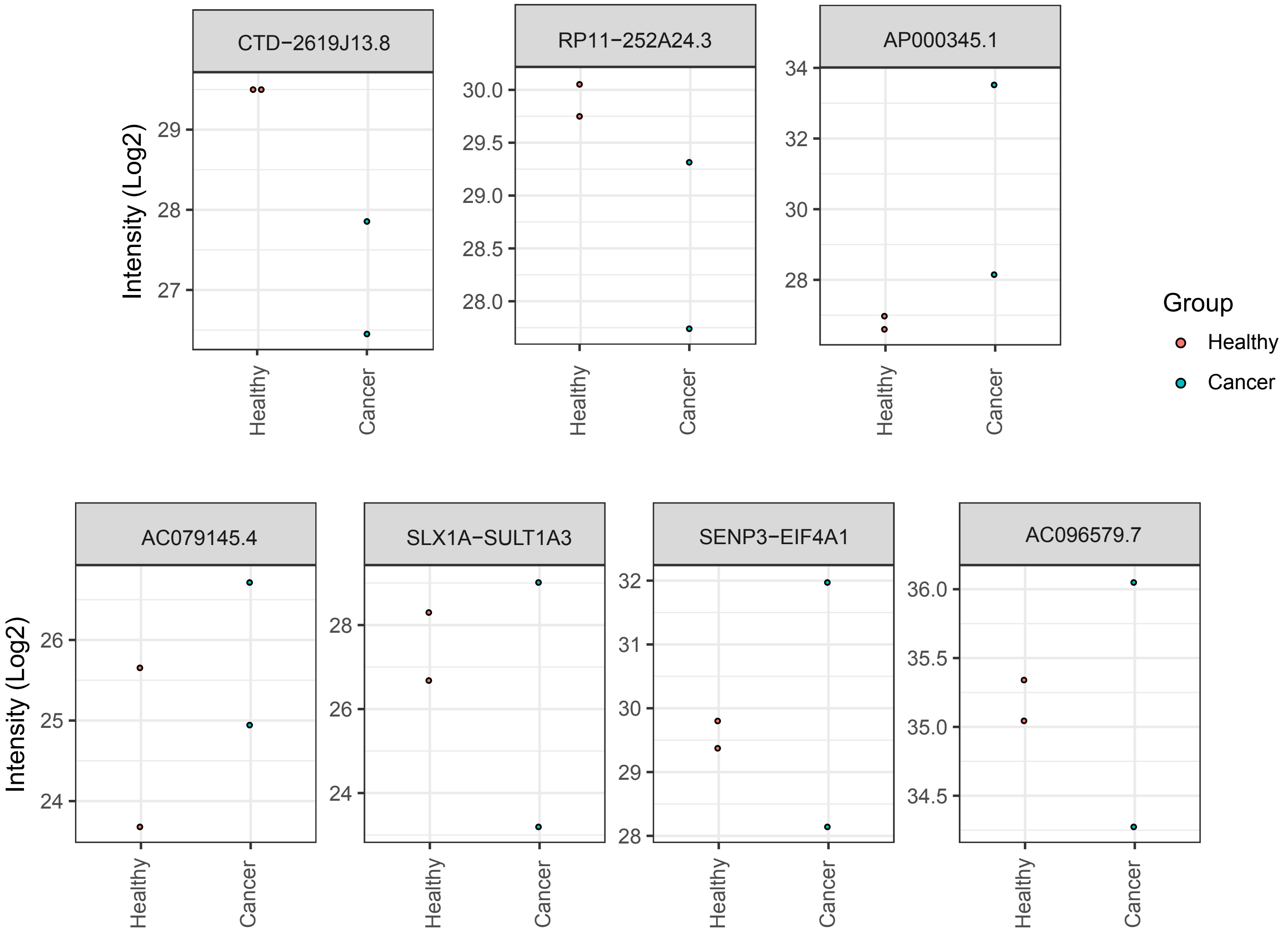

Supplementary Figure S6

A

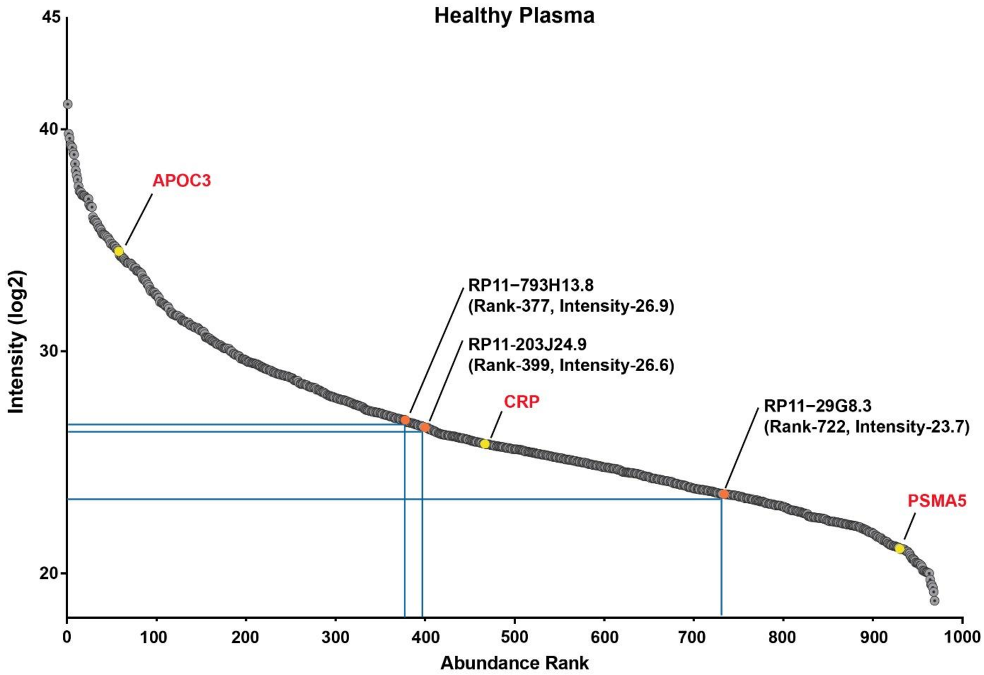

B

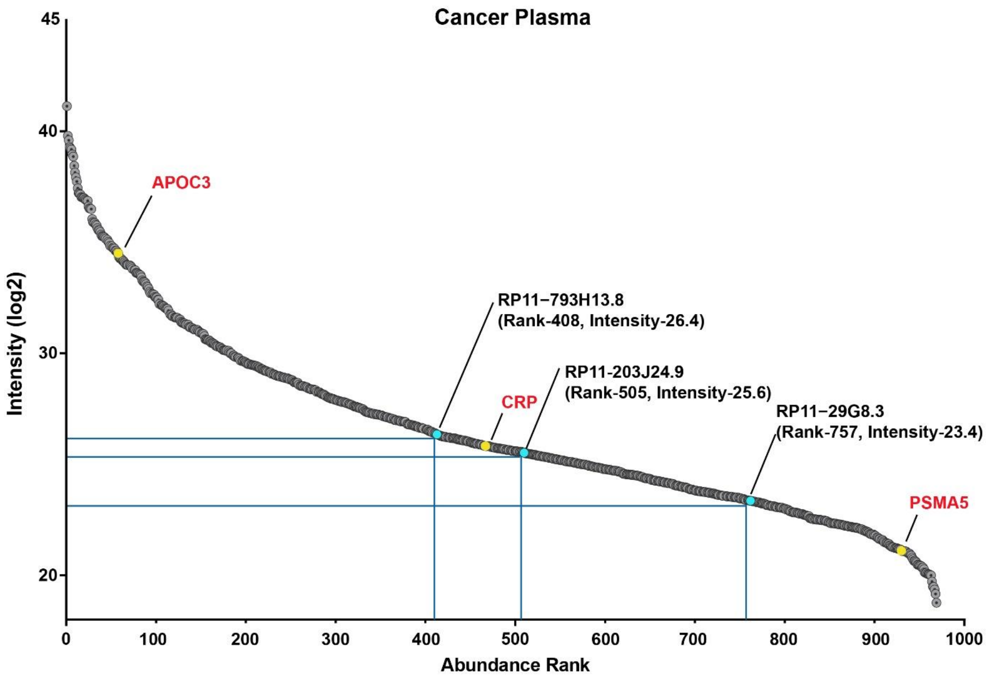

Supplement: Supplementary file 1 — Supplementary Figure 1 to 7 [file 41598_2019_48774_MOESM1_ESM.pdf]
